# RETRACTION: Surgery in Space: The Ultimate Frontier

**DOI:** 10.1113/eph.70449

**Published:** 2026-08-19

## Abstract

**RETRACTION**: M. Bashir, A. Murtada, M. Jubouri, A. Bashir, I. Williams, and D. Bailey, “Surgery in Space: The Ultimate Frontier,” *Experimental Physiology* (2025): EP092765, 10.1113/EP092765.

The above article, published online on 10 June 2025 in Wiley Online Library (wileyonlinelibrary.com), has been retracted by the Physiological Society and John Wiley & Sons Ltd., with the agreement of all authors.

Following publication, concerns were raised by a third party that one of the references in the article could not be verified. A subsequent investigation by the editors found that 12 out of 23 citations could not be substantiated as they were presented in the published article. As a result, The Physiological Society and John Wiley & Sons Ltd no longer have confidence in the article as a properly supported scholarly contribution and therefore agree that the article must be retracted.

**RETRACTION**: M. Bashir, A. Murtada, M. Jubouri, A. Bashir, I. Williams, and D. Bailey, “Surgery in Space: The Ultimate Frontier,” *Experimental Physiology* (2025): EP092765, 10.1113/EP092765.

The above article, published online on 10 June 2025 in Wiley Online Library (wileyonlinelibrary.com), has been retracted by the Physiological Society and John Wiley & Sons Ltd., with the agreement of all authors.

Following publication, concerns were raised by a third party that one of the references in the article could not be verified. A subsequent investigation by the editors found that 12 out of 23 citations could not be substantiated as they were presented in the published article. As a result, The Physiological Society and John Wiley & Sons Ltd no longer have confidence in the article as a properly supported scholarly contribution and therefore agree that the article must be retracted.

